# A deep semantic vegetation health monitoring platform for citizen science imaging data

**DOI:** 10.1371/journal.pone.0270625

**Published:** 2022-07-27

**Authors:** Asim Khan, Warda Asim, Anwaar Ulhaq, Randall W. Robinson

**Affiliations:** 1 The Institute for Sustainable Industries and Liveable Cities (ISILC), College of Engineering and Science, Victoria University, Melbourne, Australia; 2 School of Computing and Mathematics, Charles Sturt University, Port Macquarie, NSW, Australia; Tezpur University, INDIA

## Abstract

Automated monitoring of vegetation health in a landscape is often attributed to calculating values of various vegetation indexes over a period of time. However, such approaches suffer from an inaccurate estimation of vegetational change due to the over-reliance of index values on vegetation’s colour attributes and the availability of multi-spectral bands. One common observation is the sensitivity of colour attributes to seasonal variations and imaging devices, thus leading to false and inaccurate change detection and monitoring. In addition, these are very strong assumptions in a citizen science project. In this article, we build upon our previous work on developing a Semantic Vegetation Index (SVI) and expand it to introduce a semantic vegetation health monitoring platform to monitor vegetation health in a large landscape. However, unlike our previous work, we use RGB images of the Australian landscape for a quarterly series of images over six years (2015–2020). This Semantic Vegetation Index (SVI) is based on deep semantic segmentation to integrate it with a citizen science project (Fluker Post) for automated environmental monitoring. It has collected thousands of vegetation images shared by various visitors from around 168 different points located in Australian regions over six years. This paper first uses a deep learning-based semantic segmentation model to classify vegetation in repeated photographs. A semantic vegetation index is then calculated and plotted in a time series to reflect seasonal variations and environmental impacts. The results show variational trends of vegetation cover for each year, and the semantic segmentation model performed well in calculating vegetation cover based on semantic pixels (overall accuracy = 97.7%). This work has solved a number of problems related to changes in viewpoint, scale, zoom, and seasonal changes in order to normalise RGB image data collected from different image devices.

## 1 Introduction

The increasing population of the world and the change in land use by them have significantly affected vegetation and landscape composition [1, 2]. It has been discovered that changes in the kind of land cover (such as building developments) have a substantial relationship with changes in vegetation. Calculations of various vegetation indexes are frequently used to automate the identification of vegetation cover and important variations in the environment [3]. Therefore, all of these natural resources require environmental monitoring to be protected and conserved, and this is especially true for open green areas and public lands. Because vegetation plays a vital role in the improvement of an ecosystem, it enables the climate to have the positive change required for better living. This is being done with the help of land-care agencies, environmental groups, and local governments using drones, UAVs, satellites, and remote sensing.

Vegetation landscapes, by their very nature, are dynamic and constantly changing in response to human use. Unfortunately, not all changes to the landscape are positive ones. In turn, this has negative consequences for farmland and grazing resources, landscape diversity, cultural values, and biological variety [4, 5]. Vegetative landscape monitoring is a good way to make sure that changes in the landscape are going in the right direction.

It is recommended to monitor anything throughout time, which means observing and documenting any changes that occur. Monitoring is all about performing periodic assessments or surveys, collecting outcomes, and then comparing them to determine the efficacy of activities or the development of projects. Management actions might be evaluated based on the feedback they receive via the monitoring team, and it also helps to determine whether natural resources are improving, stabilising, or decreasing. Understanding how and why the land and its vegetation behave over time is essential for land managers to do their jobs effectively.

Researchers have developed a number of different algorithms for calculating the vegetation index from photographs. According to reports, researchers have placed a strong emphasis on remote sensing [6] images because of their numerous advantages, including large area exposure, reliability, and many others. The green area seen in remote sensing photos is used to extract the vegetation region available in certain places [7]. Remote sensing data is collected from above by sensors (aircraft, space) that misses the glimpse of vegetation. Accordingly, ground-level profile views are inadequate for assessing urban greenery even if green indices generated from remotely sensed image data could assist in quantifying greenery. There’s a big difference between what the average person sees on the ground and what remote sensing technologies see [8].

A simple but extremely important way of monitoring vegetation, whether it is remnant vegetation or revegetated sites, is to take a series of images, which is referred to as “photopoint monitoring” or “repeat photography” [9]. Repeat photography is a method where ground-level photographs are taken from exactly the same location at different points in time. In the case of landscapes, the time stamps between the images are usually several years or decades, sometimes even up to a whole century. However, with the technological advances in aerial and satellite remote sensing, ground-based photographs have lost most of their relevance in modern landscape monitoring. A repeat photograph is a photograph that has been purposefully created to reproduce certain characteristics of another, previously taken photograph. The new image often duplicates the location coordinates of the original, presenting the user with the identical scenario for the second time and encouraging them to compare the two images [10]. In the literature, many researchers have used repeat photography for many purposes, like estimating changes in tree lines [11], for analysing plant phenology, vegetation cover estimation [12–15] and many others.

A number of ecological studies used a combination of repeat photographs as well as field measurements to obtain quantitative results [16]. Clark et al., [17] performed point sampling along horizontal transects that were randomly placed through the image to create their results. They manually categorised each image into cover classes, and they developed the concept of image cover as a quantitative metric. Roush et al., [18] calculated the vegetation cover percentage by applying a rectangular grid on top of each photo. Fortin et al., [19] used repeat photographs collected as part of the Mountain Legacy Project to derive class-specific land cover estimates and compared them to Landsat classifications. There is one thing that all of these studies have in common: the classification step is completed manually, usually by drawing polygons around specified landscape objects and then performing a visual interpretation of the results. Despite the fact that there is a large range of automatic segmentation and classification algorithms for aerial and satellite images, there is no single best solution, but the fact that ground-based images have such an angular viewpoint means that these approaches do not operate in the same manner [20]. Rohde et al., [21] findings are based on a comparison of 100 re-photographed or “matched” historical landscape images. Changes in woody cover at every photo site were analysed and used as a proxy for climatic change in the area. Toda et al., [22], used the Bartlett Experimental Forest data to compare the phenological metrics of leaf area index, plant area index, and their associated transition dates. They gathered digital repeat photography images using two separate methods: “canopy cover” and “phenocam”. Zier et al., [23] presented his findings from an investigation of vegetation change in the San Juan Mountains conducted with repeat photography and Hendrick et al., [24] in the Appalachian Mountains. In Australia, the practise of repeat photography has been quite rare in recent years. With the help of repetitive photos, John Pickard [9], demonstrated how the landscape of Australia has changed throughout time. But most of the methods that were talked about involve a lot of manual work that takes a lot of time and does not use automation.

Digital image classification has become increasingly automated with the help of machine learning and deep learning, which has quickly become one of the most popular methodologies [25, 26]. In particular, deep learning eliminates the requirement for the time-consuming and complex feature extraction (such as fractal dimension, local binary patterns, texture features, shape features, colour features, etc.) method that was previously required. Instead, throughout the CNN training process, the model learns and extracts the necessary information on its own, without the need for human intervention. Deep learning’s most major drawback is that it necessitates the use of massive amounts of labelled training data [27]. Convolutional neural networks (CNN) are one of the deep learning architectures that are particularly well suited for image analysis because of their capacity to extract spatial characteristics from images. In convolutional neural networks, the image is fed into the network in its raw form (pixels). The network transforms the image many times. First, the image goes through many convolutional layers. In those convolutional layers, the network learns new and increasingly complex features in its layers. Then the transformed image information goes through the fully connected layers and turns into a classification or prediction. CNNs have been shown to be quite effective in a wide range of applications, including object detection [28], plant segmentation [10], classification [29–33], plant disease identification and classification [34, 35] and semantic segmentation [36–38].

Researchers have proposed various algorithms for the calculation of the vegetation index using images. However, it appears that researchers have placed a high priority on remote sensing images due to benefits such as large area exposure, repeatability, and many others [6]. Remote sensed images are used to extract the green area present in them, which represents the vegetation region available in some particular region [7]. Images from remote sensing are taken from above or from the air, which is higher than ground level and captures a larger area. This makes it hard to estimate how much vegetation there is in a certain place. Pictures taken from the ground can help a lot with figuring out how the vegetation is changing, which can be used to help the environment.

The profile view of the site can provide in-depth analysis, as demonstrated by Yang et al., [8]. They compare profile views of two different forests and demonstrate how depth information can be extracted using this technique. However, the advancement in computer vision has enabled researchers to work on the profile view images and carry out comparisons between two images taken at different times from different angles, which is basically a phenomenon of repeat photography.

Kendal et al., [39] used colour thresholding for the extraction of vegetation index. The technique proved to be promising. However, only using colour features for segmentation is not an efficient method as any clutter information in the image can match the vegetation colour. Harbaš et al., [40] used a fully convolutional network (FCN) to detect and segment roadside vegetation for the navigation of autonomous vehicles. Hung et al., [41] used a learned feature approach to classify weeds and non-weeds. The images used were acquired by an unmanned vehicle. Furthermore, in recent years, Bawden et al., [42] proposed an algorithm that classifies forbs and grass. However, researchers did not focus on the change in vegetation index using repeat photography. The major focus remained on the classification of different species, as it is really important to get information about vegetation change so that steps for the improvement of the environment can be taken.

**Fig 1:**
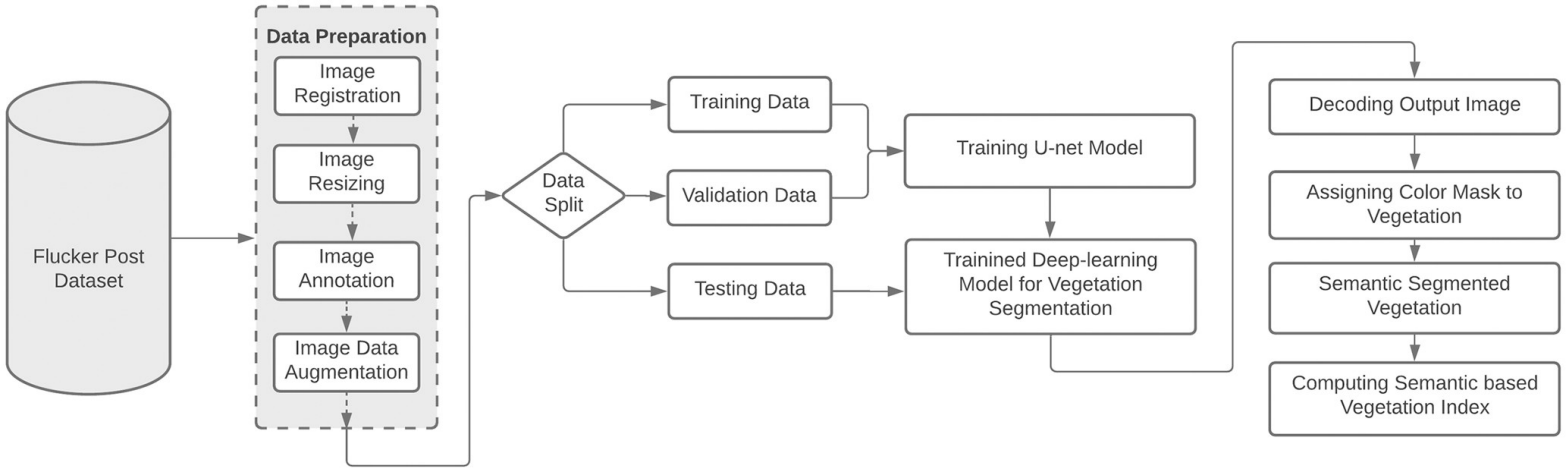
The general data flow diagram shows how the proposed system for checking the health of plants would work.

This paper focuses on the calculation of changes and monitoring in vegetation index for which registration of images is performed at an initial level. Registration is carried out because focus of authors is on repeat photography, which carries angle and scale variation. Therefore, transformation is needed to be done for this problem. This has already been done by the authors in their previously published work [43]. A novel deep affine invariant network was proposed for non-rigid image registration of multi-temporal repeated photography. Strong point matching and affine invariance are included in the suggested framework for reliable multi-temporal image registration. Furthermore, a novel approach to semantic segmentation is adopted to efficiently extract the vegetation region from the image. The vegetation region can then be used for the calculation of the vegetation index in an effective manner. The overall work flow of this proposed study is presented in Fig 1.

The rest of the paper is organised as follows: Section 2 is about the dataset taken into account; section 3 is detailed information regarding the proposed algorithm and methodology; section 4 discusses the results achieved by the proposed methodology; and section 5 is the conclusion section of this paper.

## 2 Materials and methods

### 2.1 Taking repeat photographs

It has become increasingly vital to be able to work with large numbers of images and annotate them for specific purposes. The images may come from a variety of sources, including different users, the media, artists, or even video surveillance. The images used and how they are assembled can distinguish the content and audience relevance of a work [44]. Repeat photography is more than simply taking a second picture at the same moment in time when conducting scientific research.

**Fig 2:**
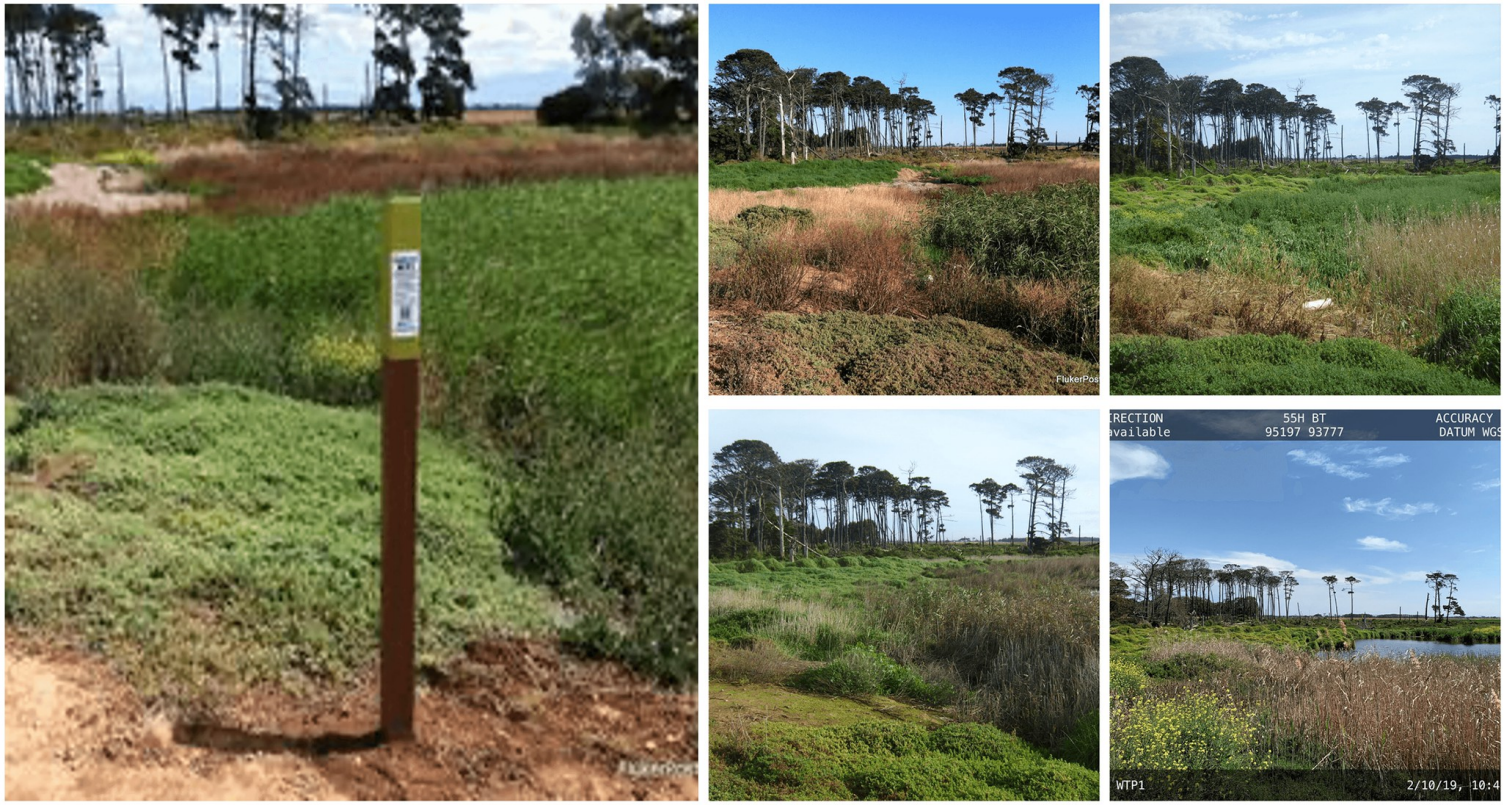
An example of repeat photography, showing images taken of a site at different times.

In order to avoid perspective shifts, these images must be taken from the same place every time. This is more important than the physical characteristics [44] shown in Fig 2. It needs careful planning and preparation, as well as close attention to the physical environment around it.

Lighting, weather, and seasonal changes should be as similar as possible in order to produce visually identical image pairs. However, weather and lighting circumstances are difficult to recreate in practise due to the fact that field activities must be planned in advance, finances are limited, and deadlines are frequently constrained in nature [45].

### 2.2 Study area / Fluker post project dataset

For the proposed technique, we used the Flukerpost dataset [46]. The Fluker Post dataset is an initiative taken by the Victorian Government and Victoria University to maintain a record of the environmental conditions of different sites throughout Australia. The dataset has been put together and is ready for people to contribute. There are around one hundred and sixty-eight (168) Fluker post point sites. At a Fluker Post point, there is no camera installed. Instead, visitors passing by from fixed photo-points use the Fluker Post app on their own phones to snap a photo of the sight in front of them. This simple method of taking pictures over and over again is a good way to manage natural resources over the long term.

**Fig 3:**
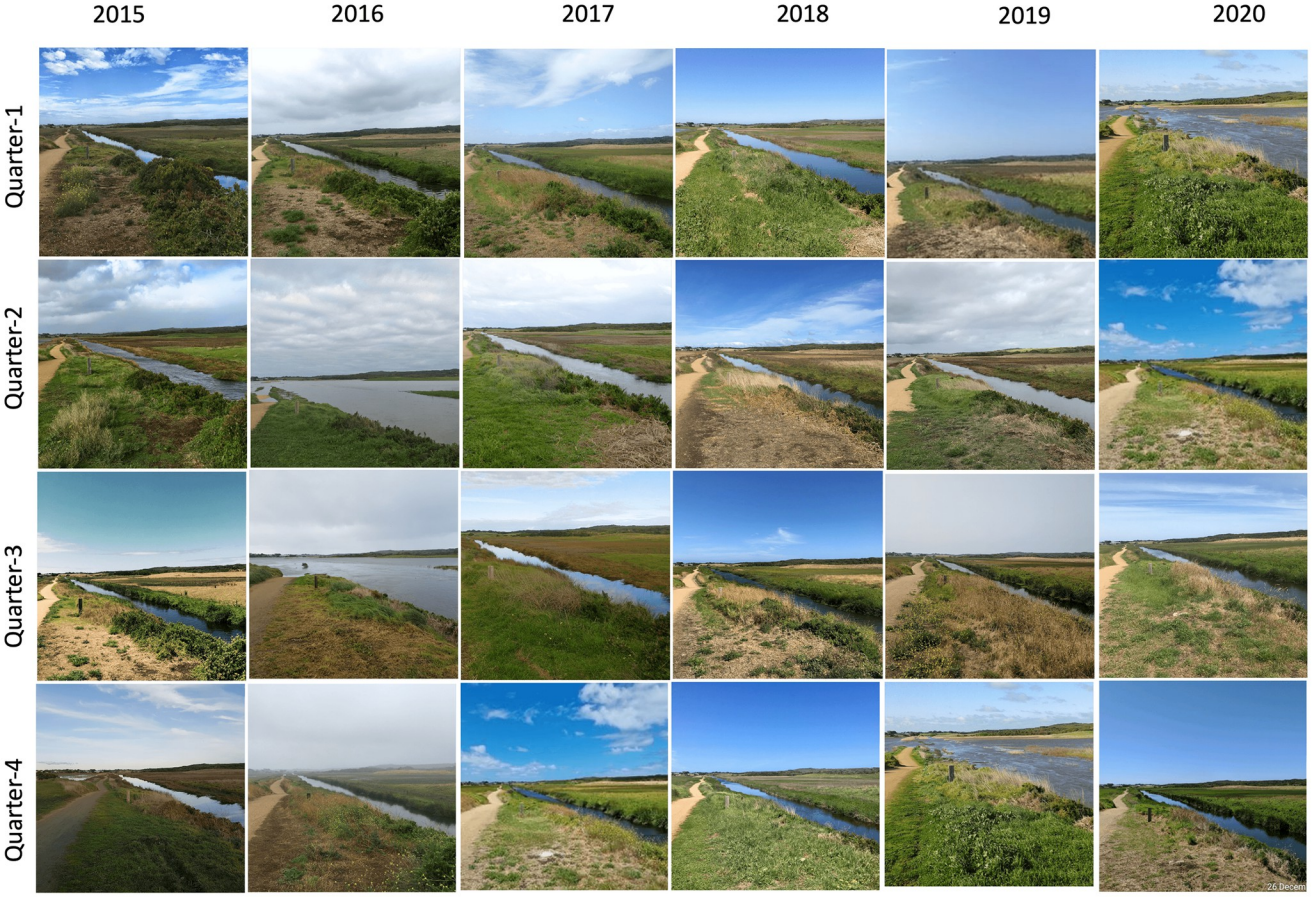
Sample images of the Warrnambool region site, one of the Fluker post points of collection, taken quarterly over several years between 2015 and 2020.

The Fluker Post has been running effectively so far, collecting useful imagery data about vegetation, parks, watersheds, rivers, streams, etc. An example of a repeated image collected from 2015 to 2020 quarterly of one study site is presented in the Fig 3.

**Fig 4:**
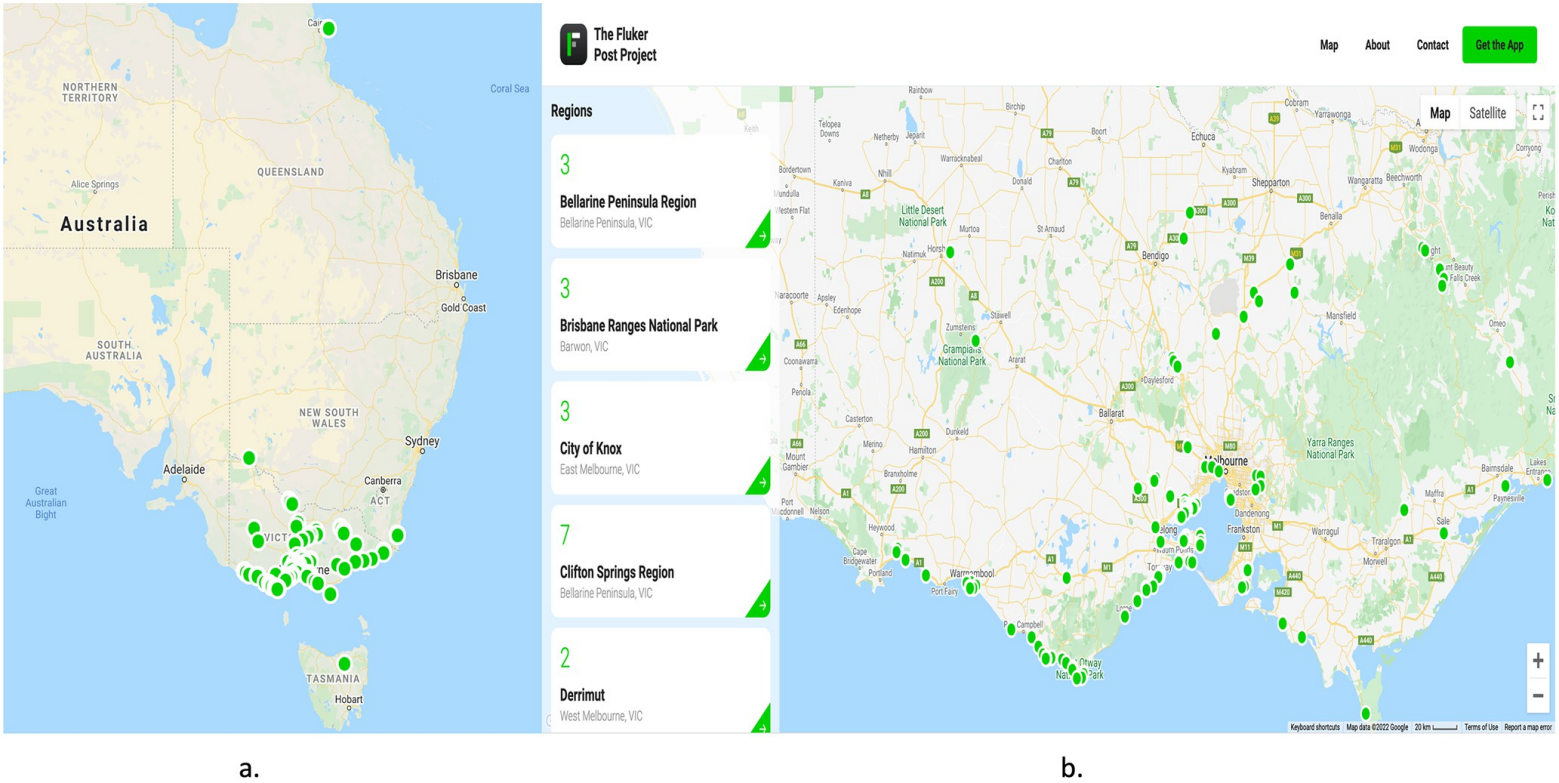
Fluker Post Project Location and Details: a.) The green circles indicate the locations of posts installed across Australia. b.) Details about each post’s location, as well as the number of images saved for a specific site.

This vast amount of photographic data is currently being manually analysed in order to point out areas where state agencies like Parks Victoria might be able to help. But because of the large volume of data, this traditional approach is less successful, and more automation is needed to improve and speed up data processing. Manual image analysis is significantly more time-consuming and inefficient when dealing with repeat photography data. In total, more than 4000 photographs have been collected for this project, all of which have been sorted into albums according to various areas throughout Australia. This project’s website address is https://www.flukerpost.com/, and its interface is shown in Fig 4. The issue, however, is that the images were captured using a variety of camera sensors and from various vantage points. Photographs are taken at random times of the day and seasons. All of these things make it hard to do, and as a result, comparing and extracting useful information like vegetation change detection has become very challenging.

### 2.3 Vegetation segmentation

The advancement of convolutional neural networks (CNNs) has achieved many milestones in the field of computer vision. They have given state of the art results for many research problems as the algorithm doesn’t require extraction of features separately and machine learning processes separately. It performs both tasks in the network and provides promising results. Networks take the image’s complex features and use them to identify any object in the image.

Our major focus is to calculate and then monitor the vegetation index of a location over different time series. For our process to be completed, we need to classify the vegetation region and non-vegetation region in the input image. We have developed a pixel-wise classification algorithm so that segmentation of vegetation can be done with the greatest efficiency.

### 2.4 U-Net

**Fig 5:**
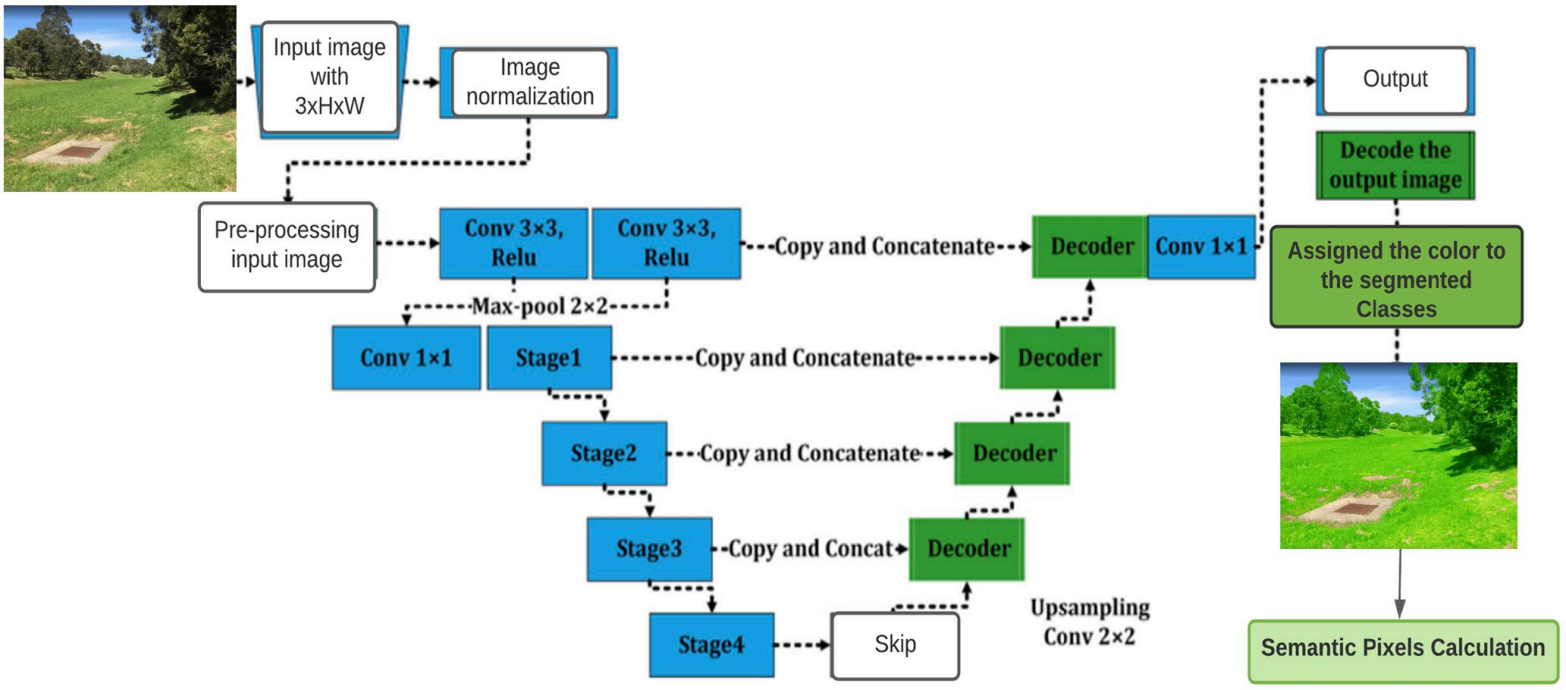
The architecture of U-Net shows network processes.

The U-Net model [47], which has an encoder-decoder architecture, as shown in Fig 5. was used in this research. The fact that U-Net is symmetric means that it does not have to deal with the connections between the up and down-sampling paths, which is advantageous when used as a concatenation operator. After they have been trained based on the colour variable in the dataset, models assign a colour to an object.

Typically, in the U-Net approach, the input image is first processed by an encoder path, which is composed of convolutional and pooling layers that degrade the spatial resolution of the input image, according to conventional practice. It is then followed by a decoder path that restores the original spatial imagery resolution by adopting up-sampling layers followed by convolutional layers, which is a technique known as “up-convolution”. Apart from that, the network makes use of so-called skip connections, which connect the output of the relevant layers in the encoder path to the inputs of the decoder path by adding them to the inputs of the decoder path.

### 2.5 Vegetation index calculation from RGB images

There are a variety of methods for calculating and monitoring the vegetation index. However, the majority of them employed either colour, threshold, or green area segmentation, which could lead to some encouraging outcomes. As a result, we propose a semantic-based approach for calculating and monitoring a robust vegetation index based on the distinctive colour of certain classes using a color-based approach. In the proposed work, RGB colour codes (107, 142, 35) were assigned to trees and vegetation terrain. After the trees and vegetation terrain have been separated, the correct masks are used to calculate the vegetation index.

### 2.6 The proposed Semantic Vegetation Index (SVI)

For robust calculation and monitoring of the vegetation index of each sample location over different time stamps, we used the approach of semantic pixels (*SP*) calculation, based on the unique colour pixels assigned to a specific class and extracted based on the deep features through the use of a deep neural network.

For vegetation index calculation and monitoring, we used the Fluker Post project image dataset, as the data is available with a time series. We observed that one image of a location is not sufficient for index monitoring. Therefore, for calculating the vegetation index in this investigation, we used the repeat photography technique. We selected four images, quarterly, per year between 2015 and 2020 for each site, out of 168 total sites. Some of the sample images of a specific site (Warrnambool Region, VIC) are shown in Fig 3. In each sample photo, the number of semantic pixels will be counted, and the area will be equal to the total number of semantic pixels in one of the three photos of a certain place.

Consequently, for the purpose of this study, a single image was used to precisely compute the vegetation index based on the semantic pixels in order to cover the whole vegetation area seen in the image, and the vegetation index was calculated using the semantic pixels approach. The number of semantic pixels in each sample image will be computed as *SP*_*a*_ with respect to the total pixels (*Area*_*t*_) of an image. The semantic vegetation index (SVI) is calculated with the following equation:

$$\begin{array}{c} SV\;I=\frac{\sum_{i=1}^{n}SP_{a_{-}i}}{\sum_{i=1}^{n}Area_{t_{-}i}}*100\% \end{array}$$ (1)

Where SVI stands for Semantic Vegetation Index, *SP*_*a*_*i*_ presents semantic pixels area in an image and *Area*_*t*_*i*_ represents the sum of pixels in an image of a specific location/site.

## 3 Experiments and results

### 3.1 Data preprocessing & preparation

The data set consists of around 3500 images taken at different times over several years. Therefore, it is essential to preprocess and prepare the data for model training in order to achieve better outcomes. We registered the images, performed data augmentation, labelled the data, and then separated the data into training, validation, and testing data (80%, 15% and 5% respectively) throughout the preprocessing and data preparation processes.

#### 3.1.1 Image registration

**Fig 6:**
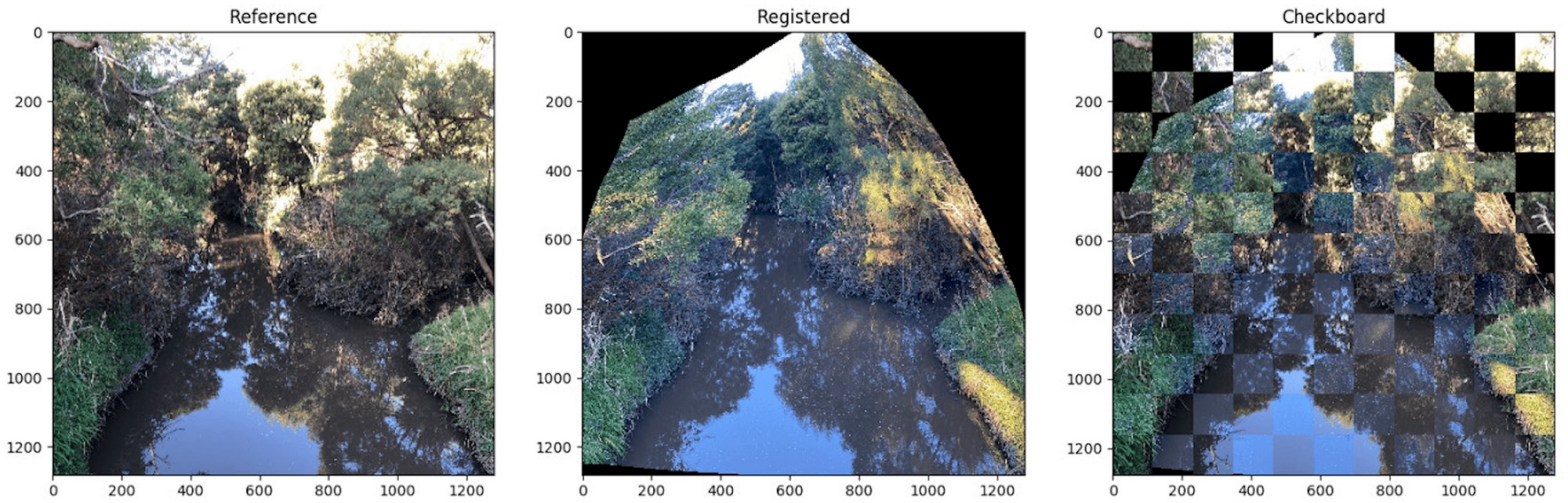
An example of image registration is applied to an input and sensed image.

Image registration is one of the most critical processes in this process. Image registration allows the system to compare two images that have undergone a similar alteration to one another. The image that is being transformed is referred to as the sensed image, and the image that is being altered in relation to it is referred to as the reference image. Image registration tries to eliminate the geometric position inconsistency between two photographs, resulting in the same image coordinates reflecting the same objects on both dates. To properly handle and analyse multiple images, it is necessary to perform image registration first. The vegetation images must be re-registered in the same dimensions as the images in the dataset because the images in the dataset were taken at various times, seasons, and locations, so their dimensions are different. To properly handle and analyse multiple photos, it is necessary to perform image registration first [48]. It’s a technique for combining photos (two or more) obtained at various times, from various vantage points, and with various sensors to create a composite image. The image registration technique is adapted from the authors’ (our) [43] previously published work. In that paper, the authors proposed a new deep affine invariant network for non-rigid image registration of multi-temporal repeat photography. Robust point matching and affine invariance are also part of the proposed framework for robust multi-temporal image registration. An example of image registration in the form of a checkerboard is presented in the Fig 6.

In this study, the images taken quarterly over a year of various site locations were used and resized to 256 x 256 pixels after the image registration process. Because the images are taken from different angles and sensors, it was necessary to use an image registration process to normalise the data for the neural network training and get better results.

#### 3.1.2 Data augmentation

**Fig 7:**
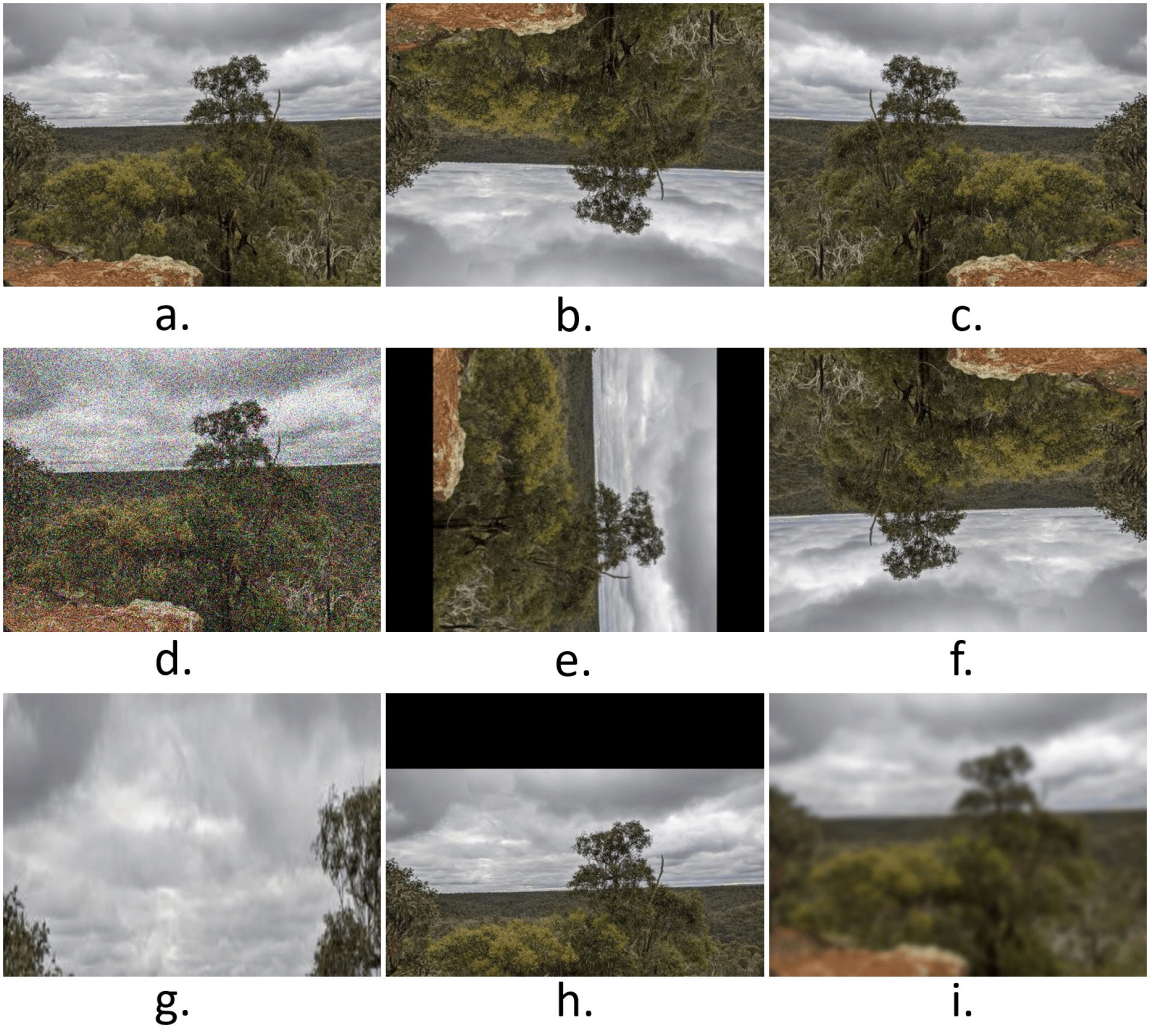
Different data augmentation technique applied include: (a). Original Image, (b). Vertical Flip, (c). Horizontal Flip, (d) Random Gaussian Noise, (e). 90 degree rotation, (f). 180 degree rotation, (g). Random zoom, (h). Translation, and (i). Blur.

A large number of images are used to train a deep neural network model to achieve highly precise prediction and accuracy. In our case, some of the Fluker post sites had fewer images than others. Because of this, the technique of data augmentation was used on the sites with fewer images. The process of data augmentation [49] provided us with new images based on our existing images. Different augmentation techniques like blurriness, rotation, flipping (horizontal and vertical), zooming, translation, and the addition of noise were applied accordingly. An illustration of different augmentation techniques is shown in Fig 7. By using this method, the number of images in our dataset grew, which is important for getting more accurate results after the training stage of a CNN.

#### 3.1.3 Data labelling

**Fig 8:**
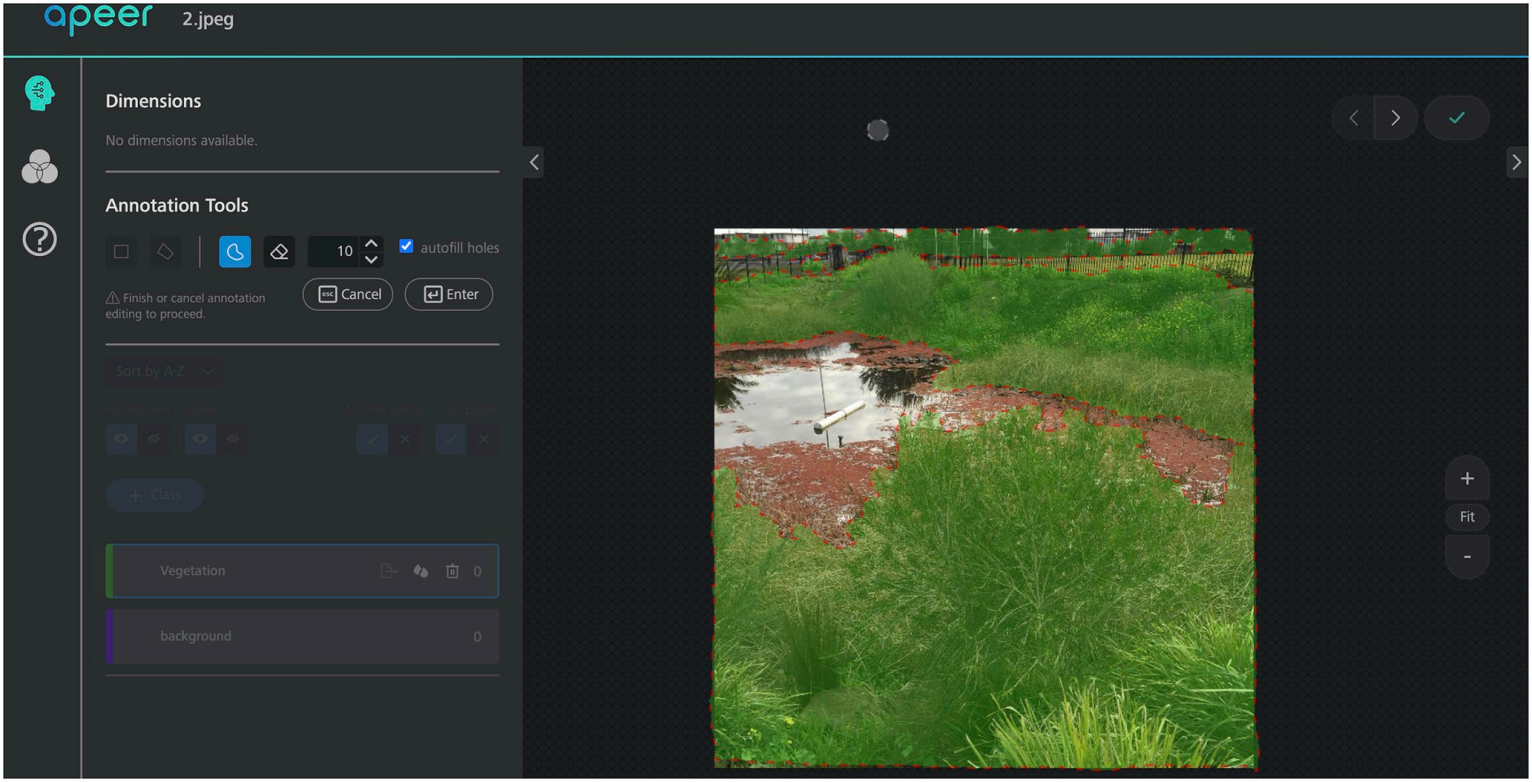
Apeer, an annotation tool’s interface, and a sample annotated image.

The light intensity of the dataset images varies since the images were taken at different times and with different cameras. Therefore, preprocessing of the dataset images is required before the registration procedure to ensure that the images are usable for the training and testing process. The training dataset was annotated with a cloud-based program called “Apeer” [50], which is available for free as part of a ZEISS initiative. Image annotation generates labels that serve as the basis for machine learning training. The amount of training data as well as the correctness of annotations are both important factors in determining machine learning accuracy. Fig 8. presents a high-level overview of the annotation process. The region of interest (ROI) is the labelled area of an image slice, which is usually only a small portion of the image. The ROI mask is inserted into the CNN with the image as a binary map, with pixels belonging to the ROI set to one and pixels belonging to the background set to zero.

### 3.2 Network model training

**Fig 9:**
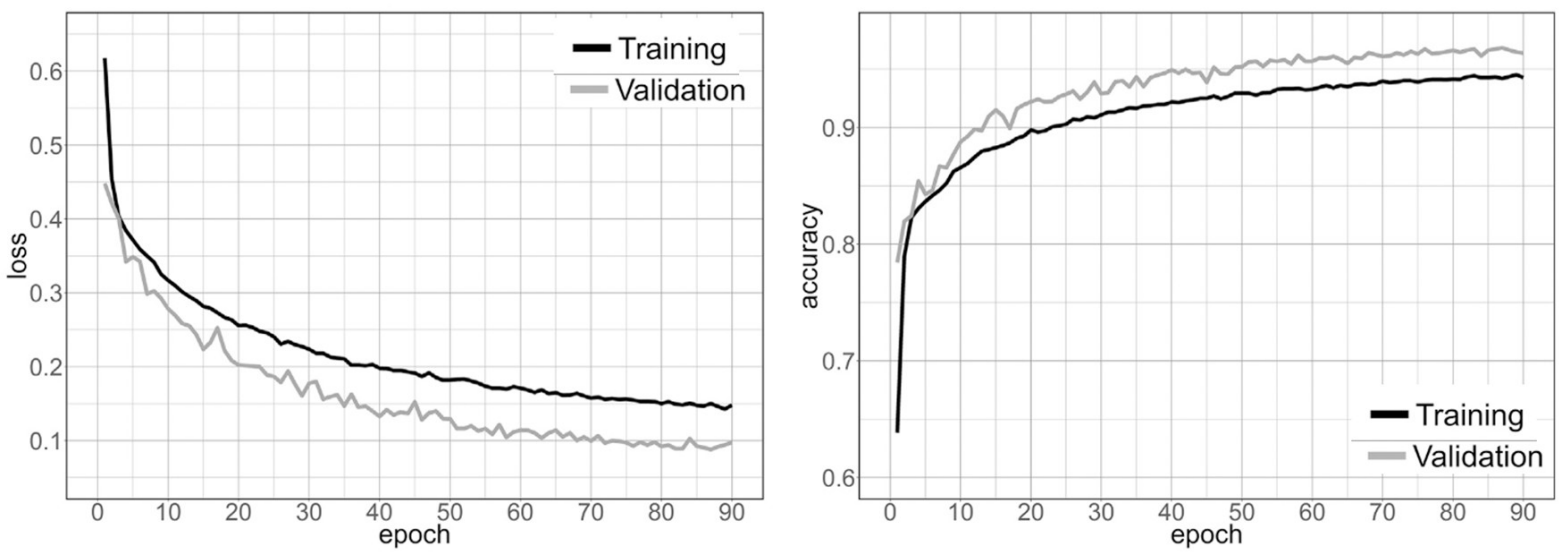
Over 90 epochs, the learning process for loss (on the left) and model accuracy (on the right) are shown. If dropout is used on the training data, the accuracy of the training data and the validation data will be different.

The entire dataset was divided into three parts: training, validation, and testing sets, each comprising 80%, 15%, and 5% of the total, respectively. Before starting the training, hyperparameters were set to avoid the overfitting and underfitting issues of the model. The hyper-parameters are set as: batch size kept at 16, learning rate as 0.0001, loss function as categorical cross-entropy, number of iterations/epochs as 200, NMS threshold as 0.45, and an optimizer as Stochastic gradient descent (SGD). The loss function ensures that the neural network optimises itself by reducing the amount of error it generates during the training process. The training loss indicates how well the model optimises the training data, while the validation loss indicates how well the model fits new data. Non-Maximum Suppression (NMS) is a technique used in numerous computer vision tasks. It is a class of algorithms to select one entity (e.g., bounding boxes) out of many overlapping entities. Most of the time, the criteria are some kind of probability number and a way to measure overlap, such as “intersection over union”. The training loss, validation loss, training accuracy, and validation accuracy curve graphs are presented in Fig 9 a.) Training and Validation Loss; b.) Accuracy of U-Net Model Training and Validation.

The Table 1 lists the hardware and software resources used in the experiments and results.

**Table 1:** The details of the configuration of the experimental environment.

Item Name	Parameter
Central processing unit (CPU)	Intel i7 9700k
Operating system	MS Windows 10
Operating volatile memory	32GB RAM
Graphic processing unit (GPU)	Nvidia Titan RTX
Development environment configuration	Python 3.8 + TensorFlow 2.5 + CUDA 11.2 + cuDNN V8.1.0 + Visual Studio 2019

### 3.3 Model performance evaluation

Both the training and validation sets were used to calculate the accuracy and loss of the model. The prediction accuracy on individual images was calculated using the 175 images from the test set (= 5%), which had been separated from the total number of samples before training. The confusion matrix is made up of pixel numbers representing true positives (*tp*), true negatives (*tn*), false positives (*fp*), and false negatives (*fn*). As for accuracy metrics, we use *Precision*(*P*), *Recall*(*R*), *F*1*score*, and *OverallAccuracy*(*OA*). These metrics are calculated as follows:

$$\begin{array}{c} Precision\left(P\right)=\frac{tp}{tp+fp} \end{array}$$ (2)

$$\begin{array}{c} Recall\left(R\right)=\frac{tp}{tp+fn} \end{array}$$ (3)

$$\begin{array}{c} F1-score=\frac{2*Precision*Recall}{Precision+Recall} \end{array}$$ (4)

$$\begin{array}{c} OverallAccuracy\left(OA\right)=\frac{tp+tn}{tp+tn+fp+fn} \end{array}$$ (5)

The learning process stopped after 90 epochs when the learning curve converged and loss values stopped decreasing. Fig 9. depicts the improvement in loss and accuracy during the training process. It is a fact, that when dropout and data augmentation are applied exclusively to training data, the validation accuracy often exceeds the training accuracy. The model achieved a maximum accuracy of 96.6% (loss = 0.11), based on the validation set. Additionally, we examined the model’s performance on a second test dataset consisting of n = 175 unique images that were not utilised in the model selection procedure. The model achieved an accuracy of 97.4% (loss = 0.07) on these individual images. After testing the model, overall accuracy varied significantly amongst images, ranging from 74.1% to 96.6%.

A comparison was also performed through two CNN architectures: Fully convolutional network (FCN) and U-Net, while keeping the same technical environments as mentioned in Table 1. After conducting experiments, the following results, as mentioned in Table 2, were achieved. A more comprehensive comparison has already been performed with the current literature studied in the author’s (our) previous published work [36].

**Table 2:** Comparative analysis of FCN and U-Net results.

Segmentation Model	Precision	Recall	F1-Score	Pixel Accuracy	IoU	mIoU
FCN	94.2	85.3	91.1	90.4	83.3	81
U-Net	96	91.8	92.3	93.4	87.5	84

**Fig 10:**
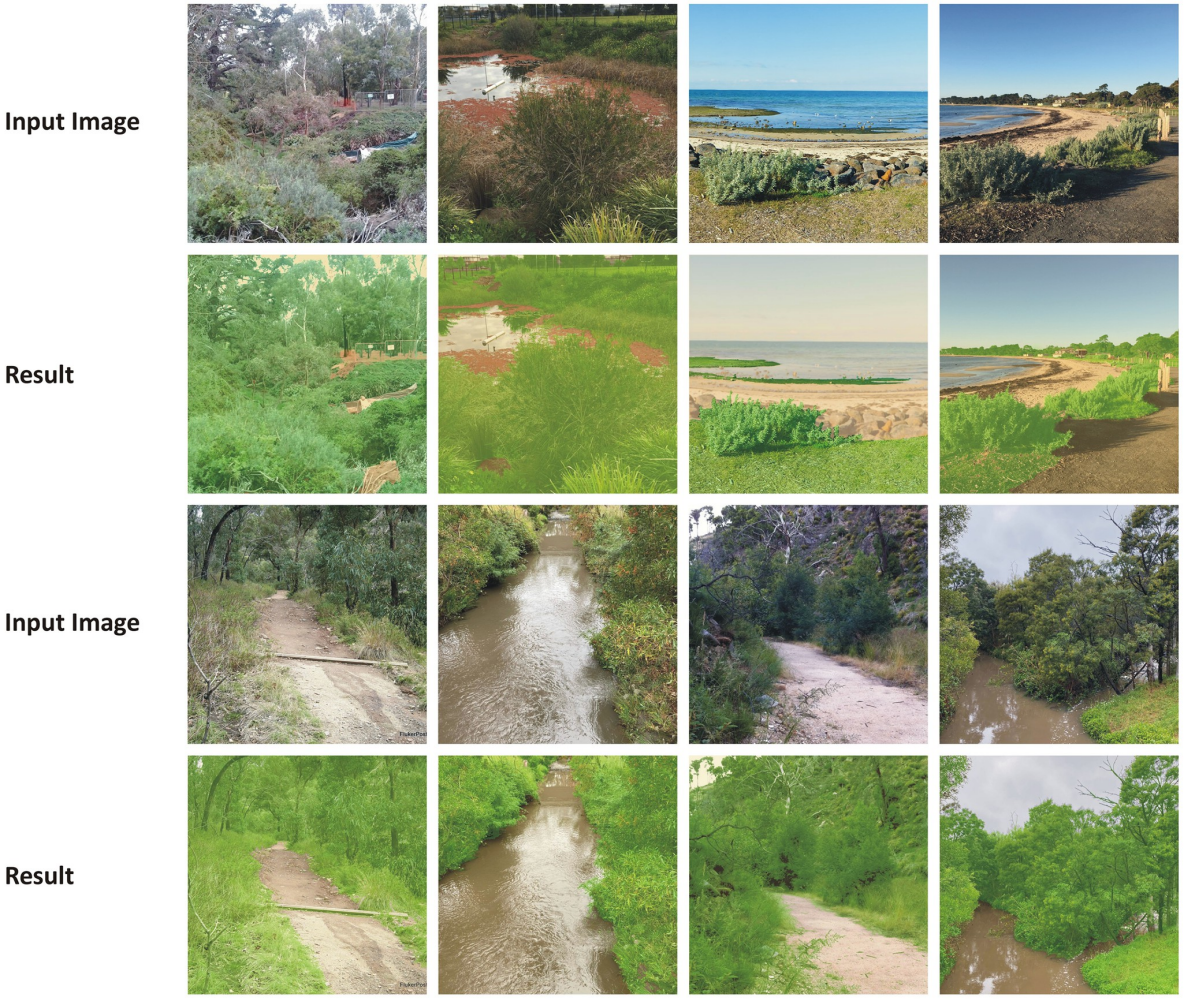
Some sample segmentation results for the randomly selected test input images.

The Fig 10. shows some of the segmentation results from the randomly selected test images. They are quite promising results.

## 4 Discussion

While evaluating the trained U-Net model’s performance in classifying vegetation in repeated landscape photographs, an over-all accuracy (OA) of 97.4% on individual images was achieved. In contrast, several studies for similar problems, such as Zhang et al., [51] used a spatial contextual superpixel model to achieve an accuracy of 79.8% on the class “tree” in real roadside images. For trees in ground images, Byeon et al., [52] used an LSTM Re-current Neural Network (RNN) to get a class accuracy of 64.2%. In a similar manner, Shuai et al., [53] paired a CNN with a directed acyclic graphic RNN (DAG-RNN) for scene identification and achieved an accuracy of 82.5% for the tree-class.

The quality of the image was recognised as a significant influencing element. It is possible that the wide range of image content, resolution, scale, and illumination used in a specific job has an impact on the classification and identification accuracy. The advancements in digital camera technology have resulted in a significant improvement in image quality over time. As a result, older RGB images are often of poorer quality than current RGB images, which has a negative impact on the efficacy of the classification and detection methods. Clark et al., [17] encounter the same image quality issues when attempting to measure vegetation changes between repeat images using transect point sampling.

When Skovsen et al., [54] attempted to differentiate clover from grasses and weeds using fuzzy images, they found a greater rate of misclassification because of the quality of the images.

**Fig 11:**
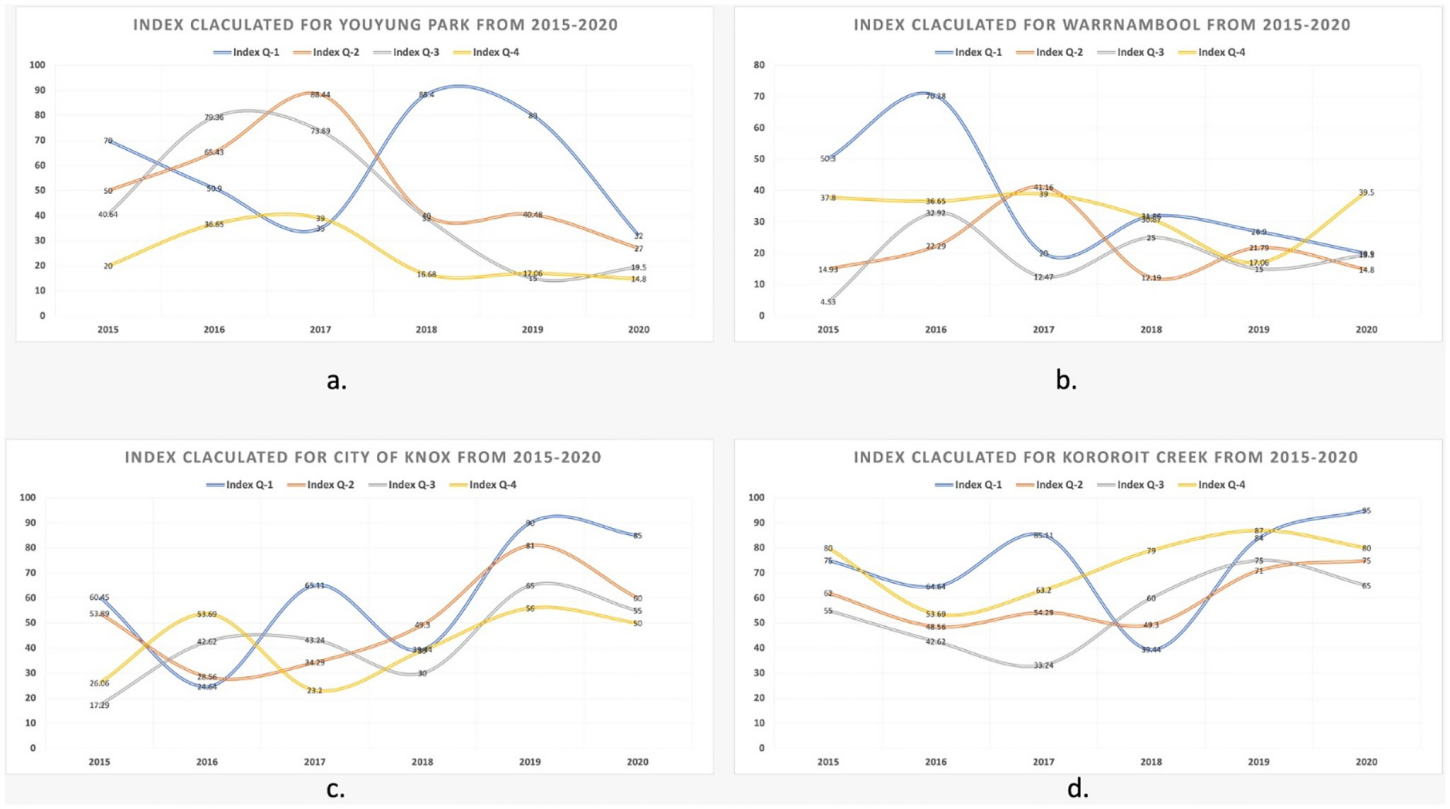
Figures depicting the average semantic vegetation index calculated quarterly from 2015 to 2020 for a.) Youyung Park, b.) Warrnambool region, c.) City of Knox, and d.) Kororoit Creek site.

Segmentation and computing the index values were done for all the Fluker Post sites. However, only a few of them (Youyung Park, the Warrnambool region, Knox, and the Kororoit Creek site) are shown in the Fig 11. From the results, it is observed that those Fluker post sites where the visitors frequently go have a large number of images for each month of the year, while some sites have fewer images. Also, there were only a couple of images for some sites. The above facts may impact the results in terms of trends. To overcome the above issue, an average index value was computed to show the trend of a specific site. For example, in Fig 11, the semantic vegetation index values computed are the average index values for each quarter of a year.

### 4.1 Seasonal variation affecting the vegetation health

According to the Bureau of Meteorology, Australia, there are several environmental factor that seriously affect the vegetation health [55]. They are:

- Low rainfalls
- Extremely dry season
- Consecutive periods of dry or cold weather
- Early and long-lasting devastating bushfires

**Fig 12:**
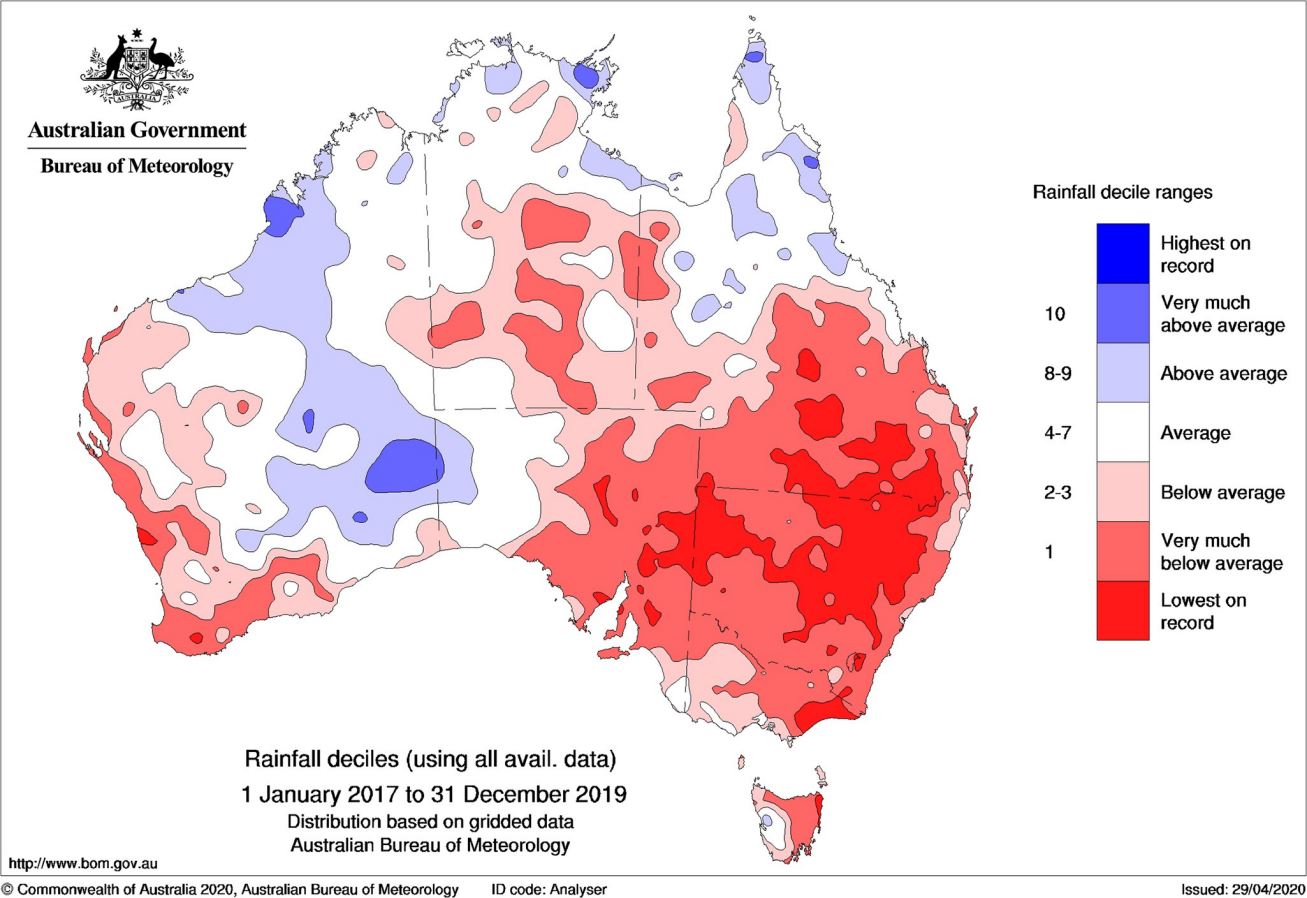
Australian rainfall deciles for the combined three-year April–September periods of 2017, 2018, and 2019. (Based on all years since 1900).

**Low rainfalls**: Australia had a very wet winter and spring in 2016. In 2017, things dried up a lot. Most of the interior of southeastern Australia didn’t get much rain in 2017, 2018, and 2019. In some places, like western Victoria and western Queensland, there was more dry weather than other parts of the country had in these years. Only a slight recovery followed a very dry and cool season in October and December 2017 and 2018. For several years, there was record-low rainfall. This year’s cool season was very dry, and it did not end until the end of the year. From January 2017 to December 2019, the Murray–Darling Basin and New South Wales have had the driest three years on record, as shown in Fig 12. Other areas that have not been getting enough rain for a long time include eastern Victoria, eastern and northern Tasmania, eastern South Australia, except for the southeast and some parts of the southwest, and Western Australia.

**Extremely dry season**: Extremely dry conditions, especially in the northern half of New South Wales, have had the worst droughts over long periods of time.This is in stark contrast to what occurred during the Millennium drought, when the southern basin had the worst droughts and the north had the best. Two other places with a long-term lack of rain, namely Gippsland in eastern Victoria and eastern Tasmania, were two others. In Gippsland, the most severe deficits were found. 2019 was marked as the third year in a row that the area did not receive enough rain. While it wasn’t as dry as in 2017 and 2018, the lack of rain kept building up over time. This led to multi-year deficits. The east coast of Tasmania also saw a lot less rain than usual during this time.

**Consecutive dry, cool seasons**: These three years (2017-2019) didn’t get enough rain, but they were terrible in the fall and winter. From 2017 to 2019, it was very dry in a lot of New South Wales from April to September each year. It was the same in Queensland, south of the Tropic of Capricorn, where the weather was the same. People in New South Wales and the Murray–Darling Basin didn’t get much rain in April and September.

**Early and long-lasting devastating bushfires**: As measured by the Forest Fire Danger Index (FFDI), which is a common way to measure fire weather conditions, spring 2019 saw the highest level of fire weather danger across the whole country. Record high FFDI values were found in all states and territories. The hot weather made things even more dangerous for fires in December 2019 and early January 2020.

**Fig 13:**
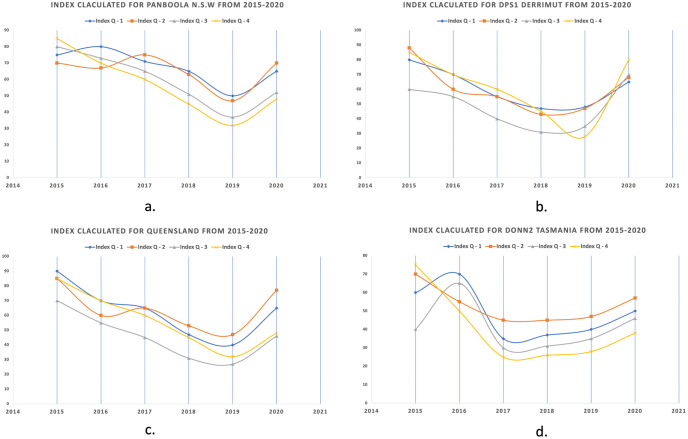
Trends of vegetation with respect to environmental factors from 2015-2020 quarterly for a.) Panboola (NSW), b.) Derrimut (VIC), c.) Queensland and d.) Donn2 (Tasmania).

As can be seen from the above trends in Fig 13, during the years 2017–2019, the environmental factors mentioned above significantly affected the vegetation in most of the Australian regions and territories. Therefore, it can be seen that the average semantic vegetation index calculated has low values due to environmental factors. However, beginning in 2020, those areas were given extra care with the help of citizens and the government to help the plants grow back and save the biodiversity.

## 5 Conclusion

This research article proposes automatic vegetation health monitoring using repetitive photography. This study tried to address challenges related to citizen science data processing. For this purpose, we normalise data collected from various visitors who visited Fluker post point sites in Australia and use it to estimate the vegetation cover change from images taken quarterly over six years in various specific locations throughout Australia. In general, using repeat photography in vegetation monitoring brings significant value to other quantitative data retrieved from remote sensing and field measurements. Furthermore, visitor-acquired photographs can raise awareness about landscape and vegetation change among policymakers and the general public and provide clear feedback on the effects of land management. It is observed that a few significant factors can impact the performance of the automatic approach, including image quality, shadow cast, and varying scale. Deep characteristics learned from a deep neural network are used to make sure that the vegetation index for each sample location is accurate. The proposed method for segmenting vegetative areas has produced encouraging results. Based on the results achieved from the semantic segmentation model, the trends are plotted. Those plotted trends revealed that some of the Fluker post site vegetation increased gradually at some locations, the vegetation treads remained almost the same, showing no significant increase or decrease, while some of the study sites showed a dramatic decline in vegetation due to floods and harsh weather. Thus, those results present the increase or decrease of vegetation at specific site locations, which can be beneficial information for agriculture management officials and the research community on a wide range of research issues. It provides a robust platform to handle citizen science data for automated community service projects. The images in the Fluker Post Project are the collection of images acquired by visitors and citizens over different times of the year with their handheld cameras and mobile devices, in other words, through various sensors. However, there are a few challenges to be kept in mind. If the same pictures were taken with the UAV, it would be important to make sure that only authorised people with licences and basic knowledge of taking pictures were doing it. Secondly, UAV equipment is expensive, so cost would be allocated for that. The same idea could be used to look at changes in vegetation in a city using Google Street View (GSV) imagery, as long as there are enough images for that city.
